# Characterizing regulatory path motifs in integrated networks using perturbational data

**DOI:** 10.1186/gb-2010-11-3-r32

**Published:** 2010-03-11

**Authors:** Anagha Joshi, Thomas Van Parys, Yves Van de Peer, Tom Michoel

**Affiliations:** 1 Department of Plant Systems Biology, VIB, Technologiepark 927, B-9052 Gent, Belgium; 2 Department of Plant Biotechnology and Genetics, UGent, Technologiepark 927, B-9052 Gent, Belgium

## Abstract

We introduce Pathicular http://bioinformatics.psb.ugent.be/software/details/Pathicular, a Cytoscape plugin for studying the cellular response to perturbations of transcription factors by integrating perturbational expression data with transcriptional, protein-protein and phosphorylation networks. Pathicular searches for 'regulatory path motifs', short paths in the integrated physical networks which occur significantly more often than expected between transcription factors and their targets in the perturbational data. A case study in Saccharomyces cerevisiae identifies eight regulatory path motifs and demonstrates their biological significance.

## Abstract 2

Pathicular – a Cytoscape plugin for analysing cellular responses to transcription factor perturbations is presented

## Rationale

When a cell is perturbed by external stimuli, it responds by adjusting the amount at which different types of proteins are needed. Transcriptional regulatory networks form the core of this cellular response system. However, the static wiring of these networks does not reveal which parts of the network are active under certain conditions and how perturbations are propagated through the network. For this reason there has been much interest in integrating the static network topology with gene expression data which reflect the dynamical or functional state of the network. In a pioneering paper, large changes were identified in the subnetworks of the transcriptional regulatory network of *S. cerevisiae *active under five different conditions [1]. In reality, the transcriptional regulatory network cannot be considered in isolation, but it is integrated with other networks such as the protein-protein interaction network [2]. In [3], a framework was developed which integrates protein-protein and protein-DNA interactions to identify active subnetworks of physical interactions in perturbational data. These subnetworks extend traditional clustering approaches by grouping genes consistent with the constraints of the physical interaction networks. In [4], a further step was taken by introducing a probabilistic model to link a causative gene, via paths in the protein-DNA and protein-protein interaction network, to the set of effect genes which are differentially expressed upon knockout of the causative gene, without requiring that the intermediate genes be differentially expressed as well. This approach was used to map DNA-damage response pathways [5] and jointly model regulatory and metabolic networks [6]. The problem to explain knockout pairs using physical interactions continues to attract much interest. In [7], an integer programming formulation was introduced and in [8] an approach based on representing the physical networks by electrical wiring diagrams was applied to the study of expression quantitative trait loci. In [9], a similar approach was used to connect genetic hits to differentially expressed genes using an integrated network containing protein-protein, protein-DNA and metabolic interactions, and in [10] a technique based on the Steiner tree problem was presented. All of these techniques have in common that they are computationally expensive and try to explain as many knockout or cause-effect pairs as possible in a particular set of experiments, but do not search for general mechanisms or path structures which are common between different (classes of) knocked-out genes.

A much simpler method was used in [11]. There all paths of length two in an integrated protein-protein and protein-DNA interaction network connecting a transcription factor to its knockout gene set were kept to study the effect of redundancy between paralogous transcription factors in perturbational data. The optimal path length was determined by a hypergeometric test between the knockout set and the set of genes reached by paths of a given length [11].

In this paper we present an alternative strategy for elucidating response-to-perturbation mechanisms in integrated networks which is based on the notion of a path-like network motif. Standard network motifs are small subgraphs which occur significantly more often in a network than expected by chance and characterize its static properties [12,13], forming functional modules in integrated networks [14]. Recently, it has been shown that by overlaying functional data over static network structures additional types of network motifs can be discovered [15]. The kind of motifs studied in [15] are so-called *activity motifs*, short patterns of timed gene expression regulation events occurring significantly more often than expected by chance in the metabolic network of *S. cerevisiae*. In the same spirit, we define *regulatory path motifs *as short, significantly enriched paths in integrated physical networks which connect a causative gene (for example, a transcription factor) to a set of effect genes which are differentially expressed after perturbation of the causative gene. Enrichment of a regulatory path indicates that it connects significantly more true cause-effect pairs than suitably randomized cause-effect pairs.

Our method is implemented as a Cytoscape [16] plug-in 'Pathicular' to identify regulatory path motifs in integrated networks. As a case study, we used comprehensive microarray data sets for 157 transcription factor deletion experiments [17] and 55 transcription factor overexpression experiments [18] in *S. cerevisiae*, together with large-scale networks of transcriptional regulatory interactions [19,20], protein-protein interactions [21] and phosphorylation interactions [22]. Our algorithm identified eight regulatory path motifs, of which five were enriched in both deletion and overexpression data. These eight motifs explain 13% of all genes differentially expressed in deletion data and 24% in overexpression data, a more than five- to ten-fold increase compared to using direct transcriptional links only, confirming that perturbational microarray experiments contain mostly indirect regulatory links. We further observed that regulatory path motifs are organized into modules of genes connected to a transcription factor by the same path and the same intermediate nodes. Perturbed targets forming such modules tend to be highly coexpressed and functionally coherent and we have used this property for predicting periodic genes and associating novel functions to genes. Finally, we considered two condition-dependent data sets, one containing deletion experiments for 27 transcription factors under DNA-damage condition [5], and one cell cycle specific data set by selecting only the cell cycle regulators from [17], and compared the relative abundance of each path motif between those data sets.

The current version of Pathicular supports functions to calculate regulatory path significance values for user-defined cause-effect and directed or undirected physical interaction networks, to visualize regulatory paths on the integrated interaction network, and to extract and visualize regulatory path modules. Pathicular is freely available for academic use.

## Results

### Direct transcriptional links in perturbational data

Perturbational expression data can be viewed as a network where each transcription factor is connected to the genes that are differentially expressed after deletion or overexpression of the transcription factor.

**Figure 1:**
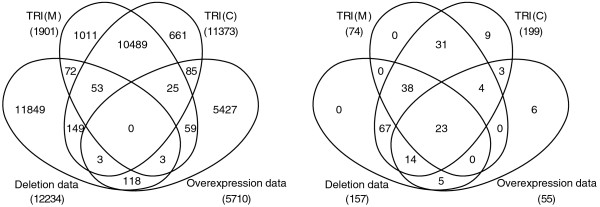
**Overlap between transcription factor-target pairs**. The overlap between four data sets of transcription factor-target pairs (**a**) and transcription factors under study (**b**) showing that there is not a single common transcription factor-target pair inferred by all methods despite 23 common transcription factors.

In [23], the topological properties of the deletion and overexpression network were compared with a transcriptional network of genome wide ChIP-chip interactions (TRI(C)), assuming that the deletion and overexpression network also consist of direct interactions. We added a fourth transcriptional network to the comparison predicted using cis-regulatory elements (TRI(M)). These four networks contain targets for 23 common transcription factors, but they do not share even a single transcription factor-target pair, although the overlap between each pair of networks is statistically significant (Figure 1). There is much higher overlap between TRI(C) and TRI(M) compared to all other pairwise combinations. On the other hand, the overexpression and deletion networks share only about 2% of their interactions with TRI(C) and TRI(M). This indicates that the deletion and overexpression networks do not contain a large fraction of direct targets.

We further calculated the overlap between each of these networks for each transcription factor individually (Table S1 in Additional File 1). Consistent with the global analysis, 18 transcription factors of 23 have significant overlap between TRI(C) and TRI(M). There is a relatively small overlap of 12 transcription factors between the deletion and overexpression network, but it is known that the deletion and overexpression phenotypes are quite different for most genes [24]. Only seven transcription factors (INO2, GCN4, SWI4, SKN7, HAP4, YAP1 and SOK2) in the overexpression network, and four (SIP4, PUT3, RFX1, MSN2) in the deletion network, share significant targets with TRI(C), without any overlap between these two sets. The seven overexpression transcription factors mainly act in response to certain conditions, for instance INO2 is activated in response to inocitol depletion and YAP1 is activated in H_2_O_2 _stress. It has been argued that overexpressing a transcription factor mimics the condition of transcription factor activation in response to a stimulus [18]. We also observed that five of these seven transcription factors (INO2, GCN4, SWI4, HAP4 and YAP1) show significant pairwise coexpression with their targets. This suggests that the overexpression method is better suited for direct target prediction of transcription factors which are activated in response to a particular signal. Similar results are obtained by comparing the overexpression and deletion networks to TRI(M).

### Indirect regulatory paths in perturbational data

When a transcription factor is deleted or overexpressed, the perturbed genes can be put into two classes. Primary targets are transcriptionally regulated by the transcription factor under study, either directly or through a regulatory cascade, while secondary targets are differentially expressed in response to the altered physiology of the cell, involving more than just transcriptional regulatory interactions. In the previous section we showed that the primary, direct targets actually form a minority in the total perturbed set. To examine the indirect modes of regulatory signal transfer, we considered an integrated physical network consisting of direct transcriptional interactions derived from ChIP-chip data (TRI), protein-protein interactions (PPI) and phosphorylation interactions (PhI). We searched in this network for regulatory path motifs, paths in the integrated network of length up to three occurring significantly more often than expected by chance between a transcription factor and its targets in the overexpression and deletion network.

### Estimating statistical significance

To assess the statistical significance of a regulatory path, we randomly permuted perturbational data (deletion and overexpression data) while keeping the number of perturbed genes for each transcription factor constant. We then compared the number of instances of a regulatory path in the integrated physical network between the real perturbational data and an ensemble of 10,000 randomized perturbational data sets. This randomization method which shuffles the expression data while keeping the wiring of the physical network intact is similar to the approaches used in [3,15].

**Figure 2:**
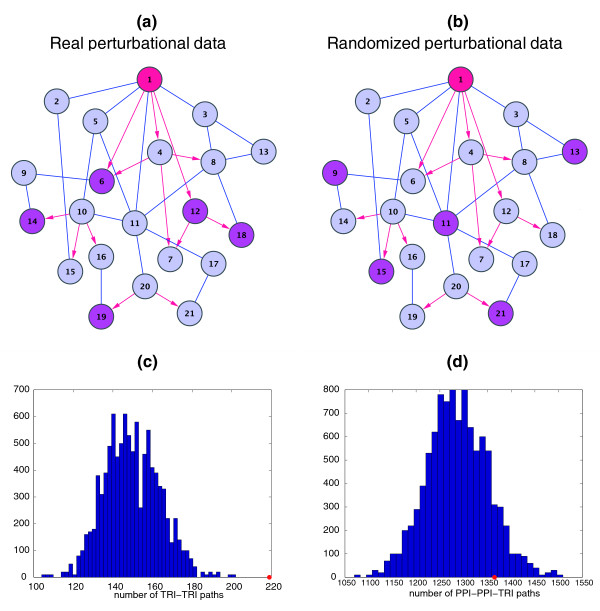
**Randomization procedure**. (**a**), (**b**) shows a hypothetical example of an integrated network of transcriptional links (red), with nodes 1, 4, 10, 12 and 20 being transcription factors, and protein-protein interactions (blue) with one hub protein (node 11). Observed perturbed genes (magenta) when a transcription factor is deleted or overexpressed (node 1, red) is shown on the left (a) and a randomized perturbed data set with the same integrated network is shown on the right (b). With respect to the background distribution of 10,000 such random samples from real data, the TRI-TRI regulatory path (**c**) is overrepresented as the observed value (red dot) lies at far right tail of the distribution (green curve), while the PPI-PPI-TRI regulatory path (**d**) is not overrepresented as the observed value lies well within the random distribution.

Figures 2a, b show a hypothetical example of an integrated network with transcriptional (red) and protein-protein interactions (blue). There are five perturbed genes (magenta) when a particular transcription factor (node 1, red) is perturbed (Figure 2a). One randomization instance is shown in Figure 2b, where the same number of perturbed genes (five in this case) are randomly assigned (magenta) while keeping the integrated network intact. This procedure is repeated to obtain 10,000 random samples. Figure 2c shows the histogram of the number of TRI-TRI paths in the randomized yeast data. The fact that the real number of TRI-TRI paths (red dot) lies at the far right of the distribution makes it a significantly enriched path. Similarly the PPI-PPI-TRI path is not observed to be enriched (Figure 2d). We compared this randomization strategy for estimating the statistical significance of a regulatory path to an alternative method based on randomizing the physical networks, and found that both are consistent (see Table 1). The alternative method keeps the perturbational data unchanged but generates random physical networks under the constraint that the distribution of outgoing and incoming paths for a particular regulatory path is constant for each node. This method extends the usual network randomization method which keeps the in- and out-degree distribution fixed. More details are given in the Methods and Additional File 1.

**Table 1:** Enrichment *P*-values for overrepresented regulatory path motifs. Enrichment *P*-values for overrepresented regulatory path motifs in deletion and overexpression data with the two randomization methods described in the Methods. The complete tables for all 39 paths can be found in Tables S4 and S5 in Additional File 1).

Regulatory path	Perturbational data randomization	Physical network randomization
	Deletion data	

TRI	7.3 × 10^-38^	4.08 × 10^-34^
TRI-TRI	6.95 × 10^-7^	1.41 × 10^-4^
TRI-PPI	5.66 × 10^-3^	1.77 × 10^-2^
PPI-TRI	1.96 × 10^-17^	1.87 × 10^-10^
PPI-PhI-TRI	8.10 × 10^-3^	1.4 × 10^-3^
PPI-TRI-TRI	2.01 × 10^-3^	2.1 × 10^-3^

	Overexpression data	

TRI	1.1 × 10^-13^	2.46 × 10^-9^
TRI-TRI	2.74 × 10^-7^	2.02 × 10^-2^
PPI-TRI	1.21 × 10^-7^	7.54 × 10^-4^
PPI-PhI-TRI	2.59 × 10^-5^	8.20 × 10^-4^
PPI-TRI-TRI	1.58 × 10^-7^	3.27 × 10^-4^
TRI-PhI-TRI	6.17 × 10^-8^	6.32 × 10^-2^
TRI-PPI-TRI	1.96 × 10^-4^	6.12 × 10^-2^

### Regulatory path motifs

**Figure 3:**
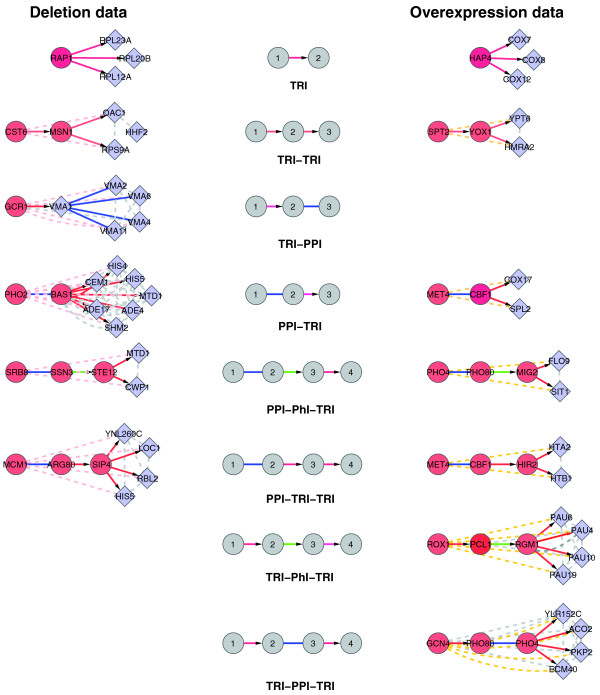
**Regulatory path motifs**. List of eight enriched regulatory path motifs in deletion and over-expression data, showing five paths common to both. Path motifs are at the center while at the sides an example in each data set is shown. TRI are in red, PPI in blue and PhI in green. The dashed gray edges represent coexpression links while pink and orange edges represent deletion and overexpression links respectively.

Out of all 39 possible paths of length up to three in the integrated TRI-PPI-PhI network, eight were significantly enriched (Table 1 and Figure 3). Five regulatory path motifs were overrepresented in both the deletion and overexpression data, namely TRI, TRI-TRI, PPI-TRI, PPI-TRI-TRI and PPI-PhI-TRI. One regulatory path motif, TRI-PPI, was overrepresented only in the deletion data, while two, TRI-PhI-TRI and TRI-PPI-TRI, were overrepresented only in the overexpression data. To check the robustness of these results, we created integrated networks obtained from different sources and using different *p*-value cutoffs (see Methods and Tables S6 and S7 in Additional File 1 for details). We also confirmed that the regulatory path motifs were not enriched because of the presence of previously well characterized overrepresented network motifs in the static network [12,13,25]. For instance, a feed-forward loop is formed by a combination of a TRI and a TRI-TRI path. We checked the enrichment of all indirect paths by removing indirect paths when also a direct path (TRI) is present, and the results still hold true. This shows that the regulatory path motifs are all significant signals independent of the simple TRI enrichment.

The enriched regulatory path motifs represent both the primary and secondary classes of perturbed targets. For instance TRI and TRI-TRI represent the direct and indirect regulatory targets, while TRI-PPI represents secondary effects. The PPI-TRI path contains transcription factors which require other transcription factors for their activity. For example, MET4 lacks DNA binding activity and requires either CBF1 or one of the two homologous proteins MET31 and MET32 for promoter association [26]. PPI-TRI-TRI extends the signal of the PPI-TRI path through another transcriptional link. We have found no simple explanation for the enrichment of the PPI-PhI-TRI path, except that it is overrepresented due to paths mainly involved in cell cycle (further discussed below). In [4], all the paths in a TRI and PPI network were found to explain differentially expressed genes, with the assumption that all paths should end by a TRI link. Overrepresentation of the TRI-PPI path shows that this assumption is not universally true. The TRI-PPI path is only enriched in the deletion network. It has been used previously for predicting novel transcription regulatory targets [27]. Since this path is overrepresented using both TRI(C) and TRI(M), we speculate that the targets of this path are not predominantly missing transcriptional links but rather the secondary response targets because of the disruption of protein complex stoichiometry.

**Figure 4:**
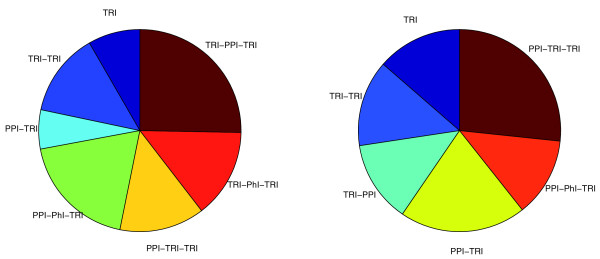
**Relative abundance of path motifs**. The relative fraction of each regulatory path motif in overexpression data (left) and deletion data (right). These show that the direct targets form a small fraction of the total number of targets.

Figure 4 shows the proportion of targets found by each regulatory path motif in the deletion and overexpression networks. It is evident that most perturbed genes are affected through indirect paths. In total, the eight enriched motifs explain 13% of all genes differentially expressed in the deletion data and 24% in overexpression data, a more than five- to ten-fold increase compared to the targets explained by direct TRI links only (see Figure 4). This leads to the conclusion that only about 10 to 20% of the perturbed genes are direct targets of the overexpressed or deleted transcription factor, a number that is in line with previous estimates. In [28] it was shown that most of the genes differentially expressed in a LEU3 mutant are not direct targets (about 20%). In [5], only 11% of deletion-buffering events (genes that are normally differentially expressed in a certain condition but become unresponsive after deleting a transcription factor) for 30 transcription factors were found to coincide with a direct ChIP-chip binding interaction. In human, only about 30 to 40% of all differentially expressed genes for NF-kB and STAT1 appear to be direct targets [29].

### Pathicular, a Cytoscape plug-in for detecting path motifs

We developed a Cytoscape [16] plug-in 'Pathicular' to identify path motifs between cause-effect pairs in integrated physical networks and to arrange them in a modular structure. The definition of the cause-effect and physical networks is up to the user. The stepwise procedure to obtain regulatory paths is as follows:

1. The cause-effect (deletion or overexpression in this case) and physical networks (transcriptional, protein-protein and phosphorylational interactions in this case) are loaded in Cytoscape.

2. A causative gene (transcription factor in this case) of interest can be selected to perform a gene-specific analysis. To perform a global analysis, all causative genes should be selected in the cause-effect network.

3. All paths of a given type are calculated by selecting the cause-effect network and physical network(s) in the 'Pathicular' panel. For each network, the checkbox should be ticked to distinguish between direct networks (which can be traversed in one direction only) and undirected networks (which can be traversed in both directions).

4. The number of random trials (default 10,000) is selected.

5. By clicking 'Execute', the program computes a *p*-value to check the overrepresentation of the path of interest and visualizes all path instances on the integrated physical network.

6. By clicking 'Modularize', the regulatory paths can be organized in modular structures for further functional analysis.

**Figure 5:**
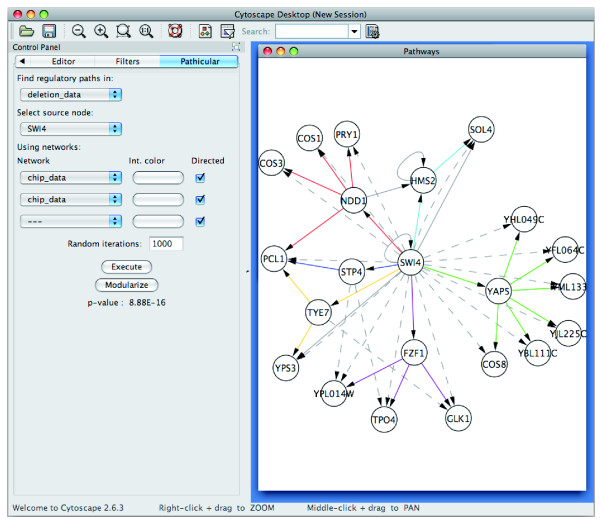
**A screenshot of Pathicular**. Screenshot of Pathicular running in Cytoscape with an example of a TRI-TRI path motif overrepresented in deletion data for the transcription factor SWI4. Solid edges represent TRI edges, colored by path module membership. Dashed edges represent edges in the deletion data. Solid gray edges are additional TRI edges which do not belong to a TRI-TRI motif in this subnetwork.

Figure 5 shows a screenshot of Pathicular with a TRI-TRI path motif overrepresented in deletion data for the transcription factor SWI4. Sample data and step-by-step instructions for running Pathicular are provided in Additional File 2.

### Comparison with other methods

Perturbational data is combined in many different ways with physical networks of protein-protein and protein-DNA interactions [3-11], see also the overview in the Background section. The approach of [3] is different from the others because it attempts to find active subnetworks of physical interactions in perturbational or condition dependent data, whereas the other methods, including ours, link causative genes to effect genes without requiring that the intermediate genes are differentially expressed. The methods of [4-10] have in common that they try to explain cause-effect pairs in a particular set of experiments by solving an optimization problem which typically balances the number of explained pairs by the length and complexity of the possible paths. These methods do not include a significance analysis with respect to randomized data and thus it is difficult to assess if a given network model truly reflects underlying regulation mechanisms or appears just by chance due to the inevitable noise inherent in the perturbational data as well as in the physical interaction networks. We illustrate this point by analyzing the output of an optimization based method [30] with our approach. More than 50% (434 of 811) of the regulatory paths predicted by [30] consist of PPI-PPI-TRI paths, but comparison with randomized data shows that this path is not overrepresented (*P*-value 0.1564). The protein-protein interaction network has a short average path length [31] and thus it is not surprising that the PPI-PPI-TRI path connects to many randomly selected genes. While undoubtedly some predicted PPI-PPI-TRI paths will be functionally relevant, the fact that many of them can be observed in randomized data puts their reliability in doubt. The advantage of our approach is that it selects only those paths which occur significantly more often than expected by chance and thus likely reflect general regulatory strategies used in biological networks, at the expense of explaining fewer cause-effect pairs overall.

In [11], also a randomization test was performed, but there it was only used to assess overall path length. More precisely they computed the hypergeometric overlap probability between the set of genes affected by a knockout and the set of genes reached by paths of a given length. As they did not consider a path specific significance test, it was found that path lengths greater than two reduced the *P*-value. However, using our approach we did find that some paths of length three are significantly enriched while not all paths of length two are significant. We verified that the significance values obtained by our randomization procedure are consistent with significance values obtained by performing a hypergeometric test as in [11] for each path separately.

### Path specificity of transcription factors

The overrepresentation of regulatory path motifs is an agglomerative effect of preference towards specific paths by all transcription factors together. We also checked the overrepresentation of each regulatory path motif for individual transcription factors. In general most perturbed targets of a transcription factor are found back with only a single path (Tables S2 and S3 in Additional File 1). The specificity of a transcription factor to a particular regulatory path is useful to characterize the mode of action of that transcription factor. For the perturbed targets of HST1 in deletion data, only the path PPI-TRI is overrepresented, where HST1 interacts with SUM1 which regulates CDA1, YFR032C, YGL138C, LOH1, BNA1 and DAL80. SUM1 and HST1 together are known to repress middle sporulation-specific gene expression during mitosis [32]. When GAL4 is deleted, it does not perturb any of its known direct targets, but the regulatory path PPI-TRI-TRI is overrepresented in its perturbed targets. For instance, GAL4 interacting with GAL3 regulates FHL1 which regulates the ribosomal genes RPL41B, RPL27B, RPS21B and RPL31B.

Other transcription factors have multiple regulatory paths overrepresented (Tables S2 and S3 in Additional File 1). MET4 is a Leucine-zipper transcriptional activator, responsible for the regulation of the sulfur amino acid pathway. When MET4 is overexpressed, 75% of the perturbed genes can be explained by the TRI, PPI-TRI and PPI-TRI-TRI motifs. MET4 regulates its target genes by working together with different combinations of the auxiliary factors CBF1, MET28, MET31 and MET32 [33] (Figure 3 shows MET4 interacting with CBF1 regulates direct targets SPL2 and COX17 and also indirect targets HTA2 and HTB1 through another transcription factor HIR2).

### Aggregation of regulatory path motifs into functional modules

Like static network motifs [13,14,34,35], regulatory path motifs aggregate into modular structures where the differentially expressed targets of a transcription factor explained by the same path through the same intermediate nodes form a module. These regulatory modules can be useful in two ways when integrated with additional data. Firstly, by integrating them with coexpression and functional data, modules validate the biological relevance of the regulatory path motifs themselves. Secondly, modules can provide better insight into the additional integrated data.

#### Coexpression and functional data

Many path modules are highly coexpressed and overrepresented in a particular functional category. We illustrate this with a few examples. The targets of PHO2 in the deletion network can be explained by a PPI-TRI path, where PHO2 interacting with BAS1 regulates HIS4, CEM1, HIS5, MTD1, SHM2, ADE17 and ADE4. All the genes in this module are mutually coexpressed and the module is overrepresented in the functional category purine nucleotide anabolism (*P*-value 9.3e-11). Another example is ROX1, a heme-dependent repressor of hypoxic genes. Its targets can be explained by a TRI-PhI-TRI path, where some of the intermediate genes are also differentially expressed in the overexpression network. A path leading to four PAU genes is especially interesting. PAU genes are known to be induced by anaerobiosis [36]. These paths predict the association of two intermediate players PCL1 and RGM1 in hypoxic stress, which is not yet studied. The regulatory path TRI-PPI is unique to the deletion network. An example of a corresponding module is given by GCR1, a transcriptional activator of genes involved in glycolysis, regulating VMA1, subunit A of the eight-subunit V1 peripheral membrane domain of the vacuolar H+-ATPase, which interacts with other proteins in this complex namely VMA2, VMA4, VMA6 and VMA11, all differentially expressed upon deletion of GCR1. The coexpression link between GCR1 and VMA1 supports the transcriptional link, while all other VMAs are neither coexpressed nor known to be transcriptionally regulated by GCR1 in TRI(C) nor TRI(M). In fact, in TRI they are known to be regulated by a completely different set of transcription factors than VMA1. This suggests that the regulation of these genes by deletion of GCR1 is performed through indirect paths in response to the disruption of protein complex stoichiometry.

Regulatory path modules can be used also for associating multiple functions to a transcription factor. SWI4 is a DNA binding component of the SBF complex which regulates late G1-specific transcription. If we calculate functional enrichment for all targets in the overexpression network, we get deoxyribonucleotide metabolism, polysaccharide metabolism, and sugar and carboxylate metabolism categories overrepresented. But by arranging them into modules we get the overrepresentation of DNA synthesis and replication, G1/S transition, mitotic cell cycle and meiosis functional categories, which explain the function of SWI4 in greater detail.

#### Prediction of periodic genes

**Figure 6:**
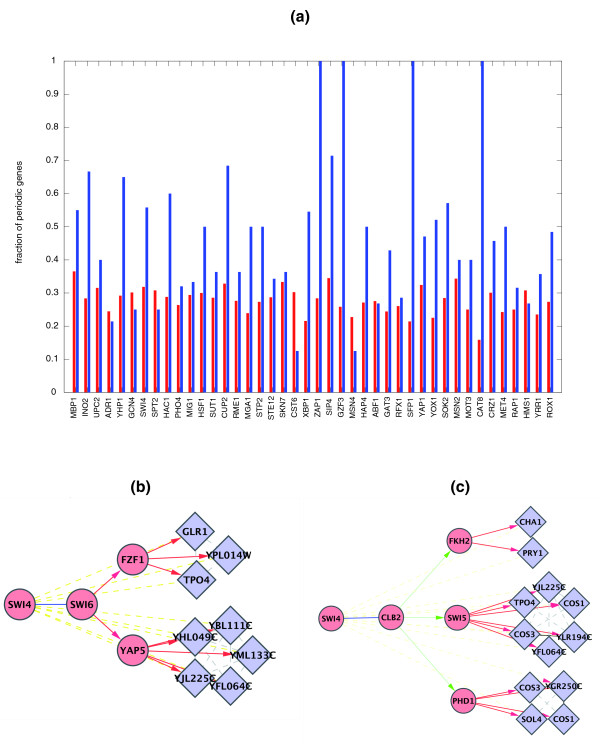
**Periodic path modules**. (**a**) The fraction of periodic targets among all perturbed genes (red) of a given transcription factor, and the fraction found in enriched motifs (blue) in overexpression data, showing all transcription factors with at least one periodically expressed target. Two path modules overrepresented in periodic genes for the PPI-TRI-TRI path in overexpression data (**b**) and the PPI-PhI-TRI path in deletion data (**c**). TRI are in red, PPI in blue and PhI in green. The dotted gray lines represent coexpression links while yellow lines represent deletion or overexpression links.

There have been four experimental efforts made to find periodically regulated genes in *S. cerevisae *[37-40]. Each one predicts a different set of genes to be periodic and assigning correct phases to periodic genes is even more difficult. There is a consensus over periodicity of only 221 genes by all experiments (Figure S2 in Additional File 1). In [41] it was shown that the information contained in the time series is not enough to establish a clear division between periodic and nonperiodic genes. As some of the regulatory path modules are also enriched in periodic genes, they can be used for predicting periodic genes and sometimes even the phase associated with them. We derived a confident set of periodically expressed genes as the ones identified in at least three experiments and considered the enrichment of periodic genes among perturbed targets of periodically expressed transcription factors. Figure 6a shows a global scenario of enrichment in periodic genes in overexpression data. A higher fraction of periodic targets is found in enriched path motifs (blue) in comparison to all perturbed genes (red). Similar enrichment is observed in deletion data (Figure S3 in Additional File 1). We illustrate this enrichment with a specific example of a cell cycle regulator, SWI4. Figure 6 shows targets of SWI4 in the deletion network reached through a PPI-PhI-TRI path (c) and in the overexpression network through a PPI-TRI-TRI path (b). We first analyzed the intermediate regulators. As transcription factors are expressed generally at low levels, it is difficult to discover periodic patterns in their expression profile. Thus FZF1 and YAP5 are not predicted to be periodic in any of the four sets mentioned above, although they are predicted to be periodic in [42]. The FZF1 targets, GLR1 (M/G1), TPO4 (G2/M) and YPL014W (M/G1), are all periodically expressed according to [38]. The phase for TPO4 was assigned G2/M in [38] while in [37] it was assigned M/G1, which matches with the rest of the genes in the module. The YAP5 targets, YBL111C (G1), YFL064C (G1), YHL049C (G1), YJL225C (M/G1) and YML133C (G1), are also all periodic and almost all peaking in expression at G1 phase. In the deletion network, a coexpressed module of six genes COS1, COS3, TPO4, YJL225C, YFL064C and YLR194C is regulated by SWI5. COS1 and COS3 are predicted to be periodic only in [40], while periodicity of the other genes is supported by at least two data sets. Thus regulatory path motifs can be used as an independent source of information for periodicity prediction. This evidence can be of more importance for lowly expressed genes like transcription factors.

### Conditional regulatory networks

**Figure 7:**
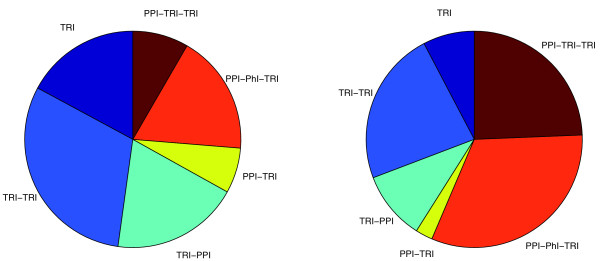
**Relative abundance of path motifs in conditional networks**. The relative fraction of each regulatory path motif in deletion data under DNA-damage stress (left) and cell cycle (right). These show differences in network structure under different conditions.

In [1], it was shown that large changes occur in the network architecture underlying exogenous and endogenous processes. More precisely, it was observed that environmental responses prefer fast signal propagation with short regulatory cascades, while cell cycle and sporulation direct temporal progression through multiple stages with highly interconnected transcription factors [1]. To see the effect of these differences on the relative abundance of each path motif, we considered two condition dependent deletion networks, one cell cycle specific and the other under DNA-damage condition (see Methods for details). In agreement with [1], in the DNA-damage network, more that 75% of the paths are of path length one or two, while the cell cycle network contains a large fraction of indirect paths with more than 50% formed by paths of length three (Figure 7).

Unlike in the DNA-damage network, about a third of the paths in the cell cycle network contain a phosphorylation link. This is not surprising since many proteins important for cell cycle progress undergo changes in their phosphorylation state during the cell cycle [43]. However, the regulatory mechanism of the PPI-PhI-TRI can be explained by literature mining only in a few cases. For instance, SWI4 interacts with CLB2 which phosphorylates FKH2. The transcriptional targets of FKH2, DSE1, PGM2 and YIL169C are perturbed in SWI4 deletion data. SWI4 binds to CDC28-CLB2 complex, which is potentially important for the regulatory activity of both proteins [44]. CDC28-CLB2 complex is capable of phosphorylating C-terminal of FKH2. This phosphorylation facilitates the recruitment of the rate-limiting transcriptional coactivator NDD1 to CLB2 and other promoters [45]. Thus the probable mechanism can be as follows. In the absence of SWI4, FKH2 is unable to form a complex with NDD1 to carry out its regulatory role. For many other PPI-PhI-TRI paths, there is no straightforward explanation. This is due to the fact that these paths are often a part of a more complex regulatory network.

## Conclusions

Genome wide expression analysis of transcription factor mutants has traditionally been used to predict novel transcription factor targets. However, as shown in this paper, these data sets contain only a small fraction (about 10 to 20%) of direct targets. In order to understand the indirect response mechanisms following the deletion or overexpression of a transcription factor, we introduced the concept of regulatory path motifs, short paths in an integrated network of transcriptional, protein-protein and phosphorylation interactions which occur significantly more often than expected by chance between transcription factors and their perturbed targets in large-scale deletion and overexpression libraries. Regulatory path motifs extend the well-known notion of static network motifs and are conceptually related to the recently introduced activity motifs. We found eight enriched paths, of which five were overrepresented in both deletion and overexpression data (TRI, TRI-TRI, PPI-TRI, PPI-TRI-TRI and PPI-PhI-TRI). The TRI-PPI path is overrepresented only in deletion data, while the TRI-PhI-TRI and TRI-PPI-TRI paths are overrepresented only in overexpression data. These eight motifs explain about 13% of all genes differentially expressed in the deletion data and 24% in overexpression data, a more than five- to ten-fold increase compared to direct transcriptional links. Like static network motifs, regulatory path motifs are organized in a modular structure where a module consists of perturbed genes reached from a transcription factor by the same type of path with the same intermediate nodes. These modules contain strongly coexpressed and functionally coherent genes and can be used for diverse purposes like predicting periodically expressed genes.

An important property of regulatory networks is their condition-dependent nature. Although currently only a limited number of transcription factor mutant expression experiments are available under different conditions, we have shown that the relative abundance of the eight path motifs in a DNA-damage and cell cycle specific network agrees well with previously observed qualitative differences between exogenous and endogenous processes. Thus regulatory path motifs can be used to characterize the condition-dependency of the response mechanisms across multiple integrated networks.

As the amount of interaction data covering cellular networks at multiple levels of regulation continues to increase, questions regarding the cross-talk between these networks and which parts of the networks are activated upon different kinds of perturbations will quickly gain importance. In this paper we have shown that searching for small, statistically overrepresented patterns integrating functional and interaction data is a simple, yet effective way to address these problems. We have implemented our method as a Cytoscape plugin Pathicular which allows to calculate regulatory path significance values, to visualize regulatory paths on the integrated interaction network, and to extract and visualize regulatory path modules.

Pathicular is applicable to a wide variety of cause-effect and physical interaction networks and is freely available for academic use.

## Methods

### Data preparation

Deletion data was obtained from [17]. We selected unfiltered interactions with a cutoff of *P*-value ≤ 0.001 (same as used by the authors). Overexpression data was obtained from [18] and the same cutoff of 0.001 was used. To find regulatory paths, we used protein-protein interactions (PPI) from [21], phosphorylation interactions (PhI) from [22] and transcriptional regulatory interactions from [19] (TRI(C)) (with a cutoff of 0.005) and [20] (TRI(M)).

### Regulatory path motif calculation

The randomization procedure applied to find overrepresented paths is as follows. For each randomization step, we randomized deletion and overexpression data by keeping the number of perturbed targets constant for each transcription factor. We then compared the number of instances of a regulatory path in the integrated physical network of TRI, PPI and PhI links between the real perturbational data and an ensemble of 10,000 randomized perturbational data sets. The physical networks are unaffected by the randomization procedure. This randomization procedure is similar to the ones described in [3,15]. We calculated the frequency occurrence of each path for 10,000 randomization steps and checked if the number of paths in the real data lies at the right tail of this distribution, using a *z*-test statistic. For small number of paths (less than five) in real data, *z*-test statistic can not be used. Significance in this case is calculated as the fraction of randomizations with number of paths equal to or more than in real data.

For the robustness analysis we checked whether the paths were significantly overrepresented in all combinations of two PPI networks, two PhI networks and four TRI networks. The two PPIs were obtained from SGD [46] and [21] and PhI data sets from SGD [46] and [22]. For transcription regulatory interactions, a ChIP-chip data set [19] with three different cutoffs 0.05, 0.005, and 0.001 on *P*-values, and targets predicted using conserved upstream regulatory motifs from [20] were used. The tables with *P*-values for all combinations of data sets are added in Additional File 1. The paths which were consistently overrepresented using all the data sets were then considered for further biological interpretation.

We furthermore compared our randomization method to another method which keeps the perturbational data unchanged but generates random physical networks such that the distribution of outgoing/incoming paths per transcription factor/target gene for a particular regulatory path is fixed. For a given path, this randomization is carried out by first creating an intermediate directed network with an edge between a transcription factor and a target if at least one instance of the path exits between this pair in the real network. This intermediate network is then randomized in the usual way by keeping its in- and out-degree distribution fixed. Both randomization methods identify the same set of enriched regulatory path motifs (see Table 1 and Tables S4 and S5 in Additional File 1).

### Periodicity

Five periodic gene prediction sets were used [37-40,42]. The list of periodic genes predicted by the authors was used. The first four sets predict also the phase while the last one does not. The confident periodically expressed gene set was produced with a criterion that the gene was identified periodic in at least three experiments. Confidence set consists of 1810 genes.

### Coexpression and functional data

The modules obtained were analyzed using two data sets:

**Coexpression data **We used a mutual information based method [47] to calculate coexpression using five large scale expression compendia of *S. cerevisiae *[38,48-51]. An adjacency matrix for each data set was created with 2.57 cutoff on the *z*-score (corresponding to 0.005 *P*-value) and these were merged to get a single coexpression adjacency matrix.

**Functional data **Functional categories from MIPS [52] were used for assessing functional coherence.

Functional overrepresentation of modules was tested using hypergeometric test.

### Conditional data

DNA-damage network for 27 transcription factors under DNA-damage stress was download from [5] while cell cycle network was generated by selecting 18 known cell cycle regulators from [17].

### Software availability

Pathicular can be downloaded from its home page [53].

## Abbreviations

PhI: phosphorylation interaction; PPI: protein-protein interaction; TRI: transcription regulatory interaction.

## Authors' contributions

AJ and TM designed the study, analyzed the data and drafted the manuscript. TVP developed the Cytoscape plug-in interface. YVdP supervised the study. All authors read and approved the final manuscript.

## Supplementary Material

Additional file 1 This pdf file contains all supplementary data for the paper. Click here for file

Additional file 2 This zip-file contains sample data and step-by-step instructions for running Pathicular. Click here for file
