# Supplementary material for: Characterizing regulatory path motifs in integrated networks using perturbational data
Source: Genome Biol. 2010 Mar 11;11(3):R32. doi: 10.1186/gb-2010-11-3-r32 (PMC2864572; doi:10.1186/gb-2010-11-3-r32)
Supplement: Additional file 1 — This pdf file contains all supplementary data for the paper. [file gb-2010-11-3-r32-S1.PDF]

Supporting online material for: Characterizing regulatory path motifs in integrated networks using perturbational data

Anagha Joshi, Thomas Van Parys, Yves Van de Peer, Tom Michoel

1 Comparison of randomization strategies

Our randomization strategy for estimating the statistical significance of a regulatory path keeps the integrated physical network fixed and randomly permutes the perturbational expression data (deletion and overexpression data) while keeping the number of perturbed genes for each transcription factor constant. We then compare the number of instances of a regulatory path in the integrated physical network between the real perturbational data and an ensemble of 10,000 randomized perturbational data sets.

The alternative method keeps the perturbational data fixed and randomly rewires the physical network while keeping the distribution of outgoing and incoming paths for a particular regulatory path constant for each node. This rewiring is carried out by creating an intermediate directed network with adjacency matrix

$$E_{ij} = \begin{cases} 1 & \text{if at least one instance of the path is present between } i \text{ and } j \\ 0 & \text{otherwise} \end{cases}$$

Then the network determined by E is rewired in the usual way by keeping its in- and out-degree distribution constant. Finally we compare the number of paths in the perturbational expression data for the real network and for an ensemble of randomized networks.

As shown in Supplementary Table 4 and 5, both randomization methods identify the same set of enriched regulatory path motifs. This indicates that our results are not dependent on the particular randomization method. Note that randomizing the physical networks requires a separate randomization for each path and is thus computationally much more expensive.

We also tested a method which randomizes each physical network (TRI, PPI, PhI) separately while keeping its in- and out-degree distribution fixed. However, this method turns out to be unsuitable for assessing the significance of longer paths since it does not preserve the number of paths per transcription factor (and not even the total number of paths for paths of length 3 or more), creating artificial biases. For instance, in randomized degree-preserving TRI networks, the number of TRI-TRI paths for a given transcription factor is simply proportional to its out-degree[1], a relation not found at all in the real TRI network (see Supplementary Figure S1). Using these degree-preserving random networks, it is found

[1] In the randomized networks, the probability for two nodes to be connected is approximately $P_{ij} \sim \frac{d_i^{out} d_j^{in}}{m}$, with d_i^{out} and d_j^{in} the out-, resp. in-degree of node i and j, and m a normalization constant. Hence the expected number of TRI-TRI paths with source i is approximately $\sum_{jk} P_{ij} P_{jk} \sim d_i^{out} \frac{\sum_j d_j^{in} d_j^{out}}{m}$.

that the number of TRI-TRI paths in the deletion data is not significantly higher in the real TRI network, although it is clear that a transcriptional cascade like the TRI-TRI path is a natural mode of regulatory signal transfer (see also the discussion in [17]).

2 Supplementary figures

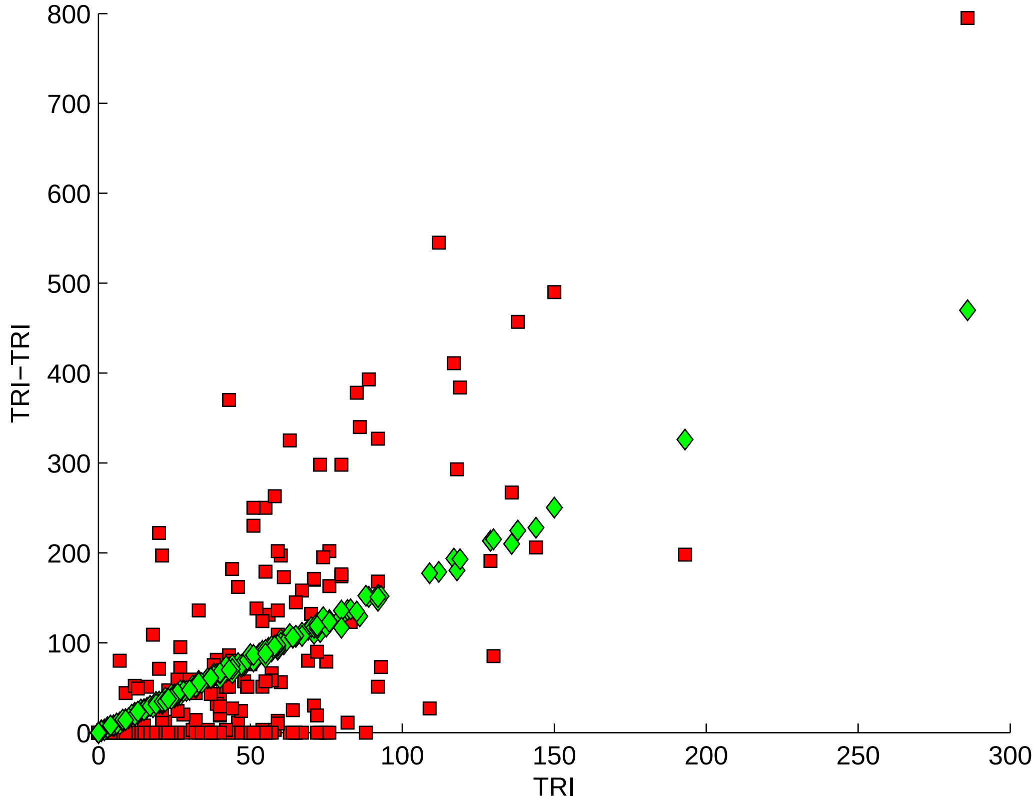

Figure S1: Relation between the TRI out-degree of each transcription factor (x-axis) and its number of TRI-TRI paths (y-axis) in the real network (red squares) and in degree-preserving randomized TRI networks (green diamonds, averaged over 1000 random networks).

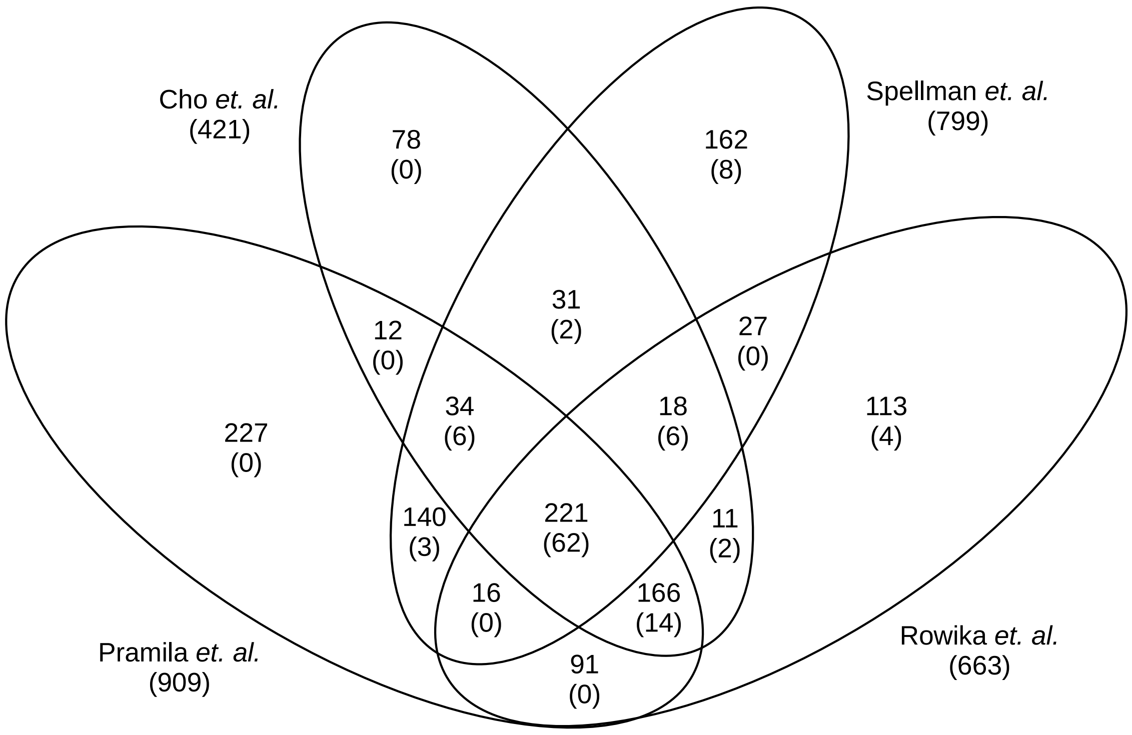

Figure S2: Venn diagram of the overlap of periodic genes predicted by 4 sets of experiments. The figure shows that there is a small overlap of only 221 genes by all methods.

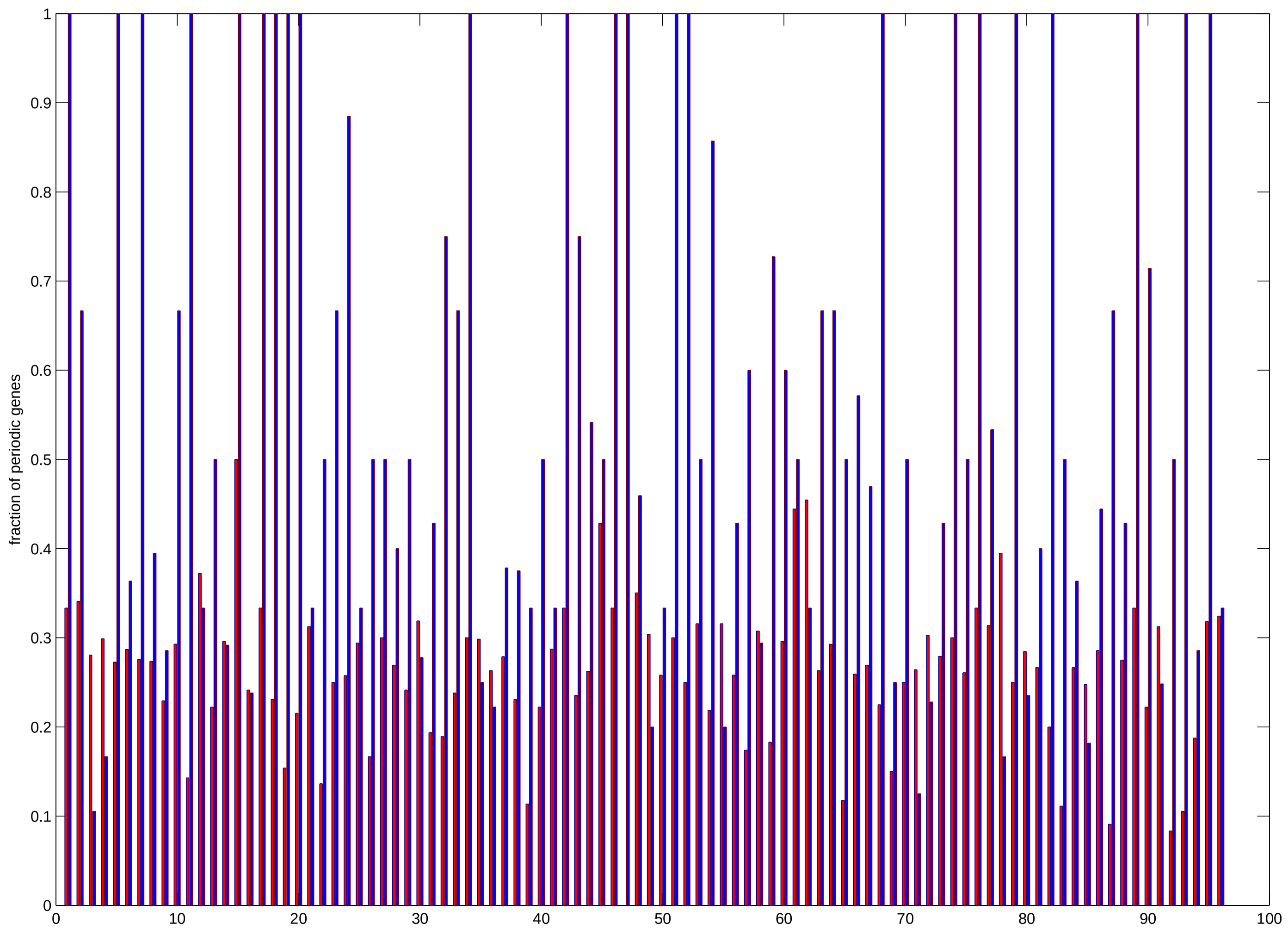

Figure S3: In deletion data, the enrichment in periodic genes in overrepresented paths with resprect to the entire network for all transcription factors.

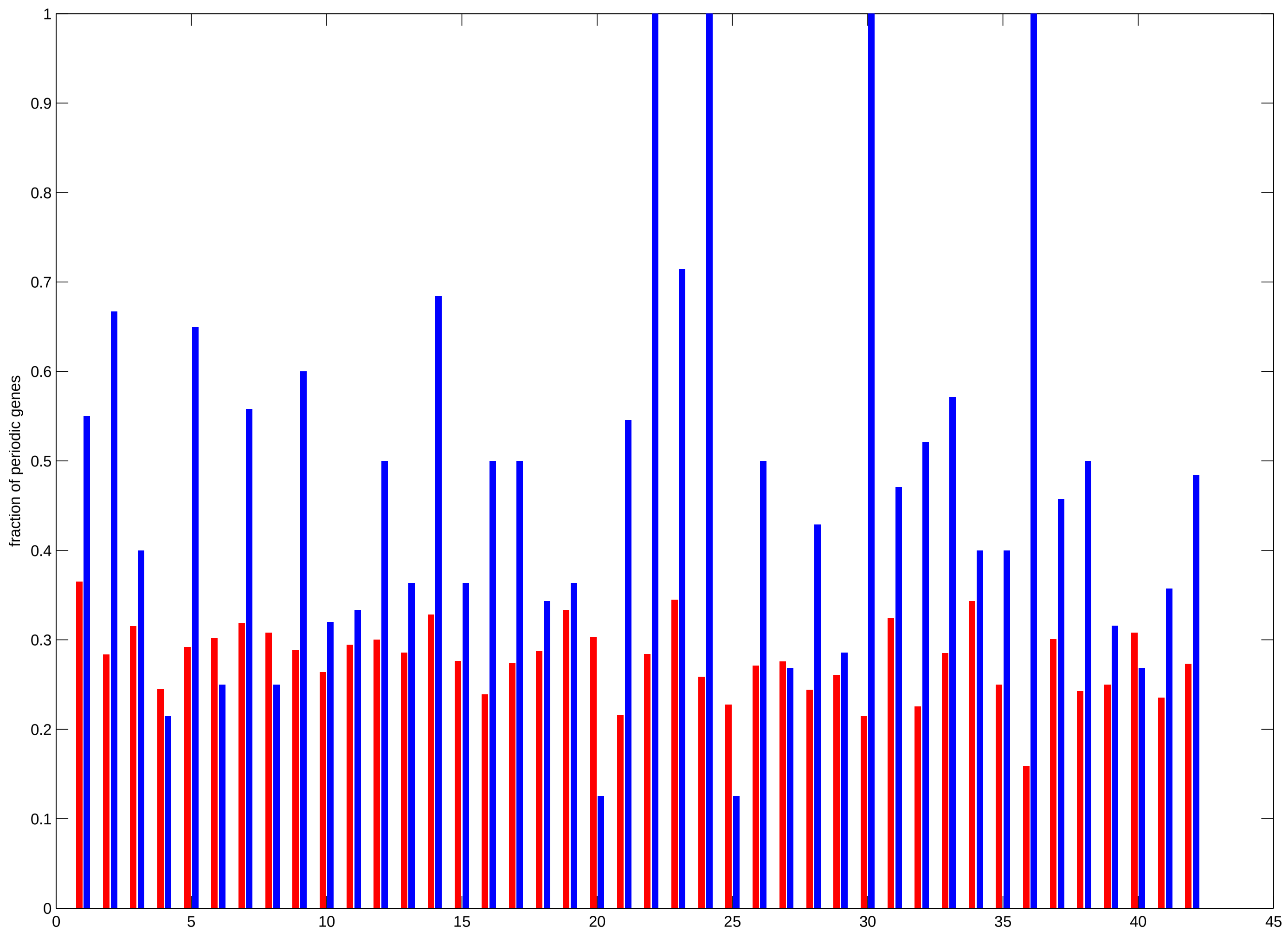

Figure S4: In overexpression data, the enrichment in periodic genes in overrepresented paths with resprect to the entire network for all transcription factors.

3 Supplementary tables

Table S1: Transcription factors having significant overlapping targets between each pair of datasets. Upper diagonal compares only 23 tf common to all datasets while lower compares all tfs present

-	TRI(M)	TRI(C)	deletion data	overexpresison data
TRI(M)	-	18	12	10
TRI(C)	59	-	4	7
deletion data	27	23	-	12
overexpresison data	12	8	22	-

Table S2: Path specificities of transcription factors in deletion data.

path	transcription factor	p value
TRI-TRI	SWI4	1.3×10^{-4}
TRI-TRI	RSF2	9.13×10^{-5}
TRI-TRI	RFX1	3.1×10^{-3}
TRI-TRI	SFP1	4.8×10^{-3}
TRI-TRI	SOK2	4.9×10^{-3}
TRI-TRI	MCM1	9.45×10^{-4}
TRI-TRI	RAP1	8.59×10^{-6}
PPI-TRI	PHO2	3.68×10^{-10}
PPI-TRI	SWI4	2.8×10^{-3}
PPI-TRI	GAL80	1.14×10^{-4}
PPI-TRI	GCR2	3.16×10^{-6}
PPI-TRI	HST1	3.73×10^{-7}
PPI-TRI	DIG1	1.35×10^{-6}
PPI-TRI	GCR1	2.5×10^{-5}
TRI-PPI	BAS1	1.6×10^{-3}
TRI-PPI	RFX1	2.22×10^{-7}
TRI-PPI	SFP1	3.26×10^{-4}
TRI-PPI	RAP1	0
TRI-PPI	GCR1	2.94×10^{-8}
PPI-PhI-TRI	SWI4	1.4×10^{-3}
PPI-PhI-TRI	SKO1	2.1×10^{-3}
PPI-PhI-TRI	DIG1	3.8×10^{-4}
PPI-TRI-TRI	SWI4	3.83×10^{-4}
PPI-TRI-TRI	GAL4	6.44×10^{-4}

Table S3: Path specificities of transcription factors in overexpression data.

path	transcription factor	p value
TRI-TRI	MBP1	1.17×10^{-4}
TRI-TRI	SOK2	4.3×10^{-3}
TRI-TRI	ROX1	1.75×10^{-4}
PPI-TRI	MBP1	3.2×10^{-3}
PPI-TRI	SWI4	1.43×10^{-4}
PPI-PhI-TRI	SWI4	1.9×10^{-3}
PPI-TRI-TRI	MBP1	4.86×10^{-4}
PPI-TRI-TRI	INO2	2.6×10^{-2}
PPI-TRI-TRI	MET4	1.91×10^{-6}
PPI-PhI-TRI	MBP1	3.42×10^{-4}
PPI-PhI-TRI	ROX1	1.61×10^{-5}
TRI-PPI-TRI	STE12	3.8×10^{-3}
TRI-PPI-TRI	ABF1	6.5×10^{-4}
TRI-PPI-TRI	YOX1	1.4×10^{-3}
TRI-PPI-TRI	MOT3	1.0×10^{-3}

Table S4: P-values for overrepresented regulatory path motifs in deletion data with two different randomization methods

regulatory path	perturbational data randomization	physical network randomization
TRI	7.3003×10^{-38}	4.08×10^{-34}
PPI	0.35381	0.36
PhI	0.67561	0.31
TRI-TRI	6.9548×10^{-7}	1.41×10^{-4}
TRI-PPI	0.0056573	1.77×10^{-2}
PPI-TRI	1.9569×10^{-17}	1.87×10^{-10}
PPI-PPI	0.16656	0.02
TRI-PhI	0.20415	0.0.57
PhI-TRI	0.0087197	0.04
PhI-PhI	0.59917	0.45
PhI-PPI	0.076439	0.12
PPI-PhI	0.10885	0.63
PPI-PPI-TRI	0.059515	0.015
PPI-PPI-PhI	0.1497	0.26
PPI-PPI-PPI	0.82528	0.47
PPI-TRI-TRI	0.002013	2.1×10^{-3}
PPI-TRI-PhI	0.67017	0.31
PPI-TRI-PPI	0.059515	0.80
PPI-PhI-PPI	0.17902	0.15
PPI-PhI-PhI	0.1946	0.28
PPI-PhI-TRI	0.008109	1.4×10^{-3}
PhI-PPI-TRI	0.44362	0.05
PhI-PPI-PhI	0.60286	0.35
PhI-PPI-PPI	0.21012	0.72
PhI-TRI-TRI	0.482	0.53
PhI-TRI-PhI	0.73301	0.75
PhI-TRI-PPI	0.74558	0.21
PhI-PhI-PPI	0.71124	0.21
PhI-PhI-PhI	0.55712	0.29
PhI-PhI-TRI	0.31186	0.21
TRI-PPI-TRI	0.7594	0.15
TRI-PPI-PhI	0.50245	0.52
TRI-PPI-PPI	0.028912	0.02
TRI-TRI-TRI	0.14744	0.12
TRI-TRI-PhI	0.23071	0.64
TRI-TRI-PPI	0.067938	0.72
TRI-PhI-PPI	0.53997	0.01
TRI-PhI-PhI	0.70691	0.09
TRI-PhI-TRI	0.19146	0.37

Table S5: P-values for overrepresented regulatory path motifs in overexpression data with two different randomization methods. '-' means no regulatory paths of a given type were observed in real data.

regulatory path	perturbational data randomization	physical network randomization
TRI	1.0955×10^{-13}	2.46×10^{-9}
PPI	0.31239	0.96
PhI	-	-
TRI-TRI	2.7434×10^{-7}	2.02×10^{-2}
TRI-PPI	0.79937	0.13
PPI-TRI	1.21×10^{-7}	7.54×10^{-4}
PPI-PPI	0.73384	0.09
TRI-PhI	0.63614	0.80
PhI-TRI	-	-
PhI-PhI	-	-
PhI-PPI	-	-
PPI-PhI	0.1523	0.64
PPI-PPI-TRI	0.85596	0.06
PPI-PPI-PhI	0.90327	0.57
PPI-PPI-PPI	0.77686	0.29
PPI-TRI-TRI	1.5824×10^{-7}	3.27×10^{-4}
PPI-TRI-PhI	0.51295	0.08
PPI-TRI-PPI	0.08037	0.10
PPI-PhI-PPI	0.21484	0.39
PPI-PhI-PhI	0.59242	0.28
PPI-PhI-TRI	2.5956×10^{-5}	8.20×10^{-4}
PhI-PPI-TRI	0.19428	0.14
PhI-PPI-PhI	0.81863	0.08
PhI-PPI-PPI	0.047571	0.98
PhI-TRI-TRI	-	-
PhI-TRI-PhI	-	-
PhI-TRI-PPI	-	-
PhI-PhI-PPI	0.91354	0.05
PhI-PhI-PhI	0.7589	0.09
PhI-PhI-TRI	0.25948	0.13
TRI-PPI-TRI	0.00019631	6.12×10^{-2}
TRI-PPI-PhI	0.15159	0.53
TRI-PPI-PPI	0.82419	0.32
TRI-TRI-TRI	0.59242	0.1
TRI-TRI-PhI	0.5114	0.33
TRI-TRI-PPI	0.21484	0.18
TRI-PhI-PPI	0.55091	0.28
TRI-PhI-PhI	0.88646	0.55
TRI-PhI-TRI	6.1744×10^{-8}	6.32×10^{-2}

Table S6: P-values for overrepresented regulatory path motifs in deletion data with different cutoffs on transcriptional data

regulatory path	TRI(C)-h	TRI(C)-m	TRI(C)-l	TRI(M)
TRI	5.4525×10^{-33}	7.3003×10^{-38}	2.6831×10^{-38}	1.1329×10^{-98}
PPI	0.33271	0.35381	0.32402	0.78298
PhI	0.73948	0.67561	0.72492	0.64319
TRI-TRI	0.00025075	6.9548×10^{-7}	1.1276×10^{-6}	3.5306×10^{-25}
TRI-PPI	9.8954×10^{-9}	0.0056573	6.9976×10^{-12}	0.062247
PPI-TRI	3.0675×10^{-15}	1.9569×10^{-17}	1.853×10^{-13}	0.033381
PPI-PPI	0.025291	0.16656	0.69464	0.3663
TRI-PhI	0.24088	0.20415	0.055277	1.0458×10^{-6}
PhI-TRI	0.043128	0.0087197	0.036763	0.21495
PhI-PhI	0.62984	0.59917	0.61358	0.58685
PhI-PPI	0.020719	0.076439	0.016102	0.42094
PPI-PhI	0.52427	0.10885	0.48342	0.4964
PPI-PPI-TRI	0.018147	0.059515	0.078665	0.62247
PPI-PPI-PhI	0.42061	0.1497	0.26761	0.073205
PPI-PPI-PPI	0.80026	0.82528	0.84004	0.78298
PPI-TRI-TRI	0.0061508	0.002013	0.0023956	0.0044328
PPI-TRI-PhI	0.66506	0.67017	0.68506	0.643194
PPI-TRI-PPI	0.018147	0.059515	0.078665	0.62247
PPI-PhI-PPI	0.0212194	0.17902	0.73657	0.3663
PPI-PhI-PhI	0.29396	0.1946	0.040307	0.10458
PPI-PhI-TRI	0.012996	0.008109	0.0014	1.9481×10^{-5}
PhI-PPI-TRI	0.39683	0.44362	0.43403	0.42094
PhI-PPI-PhI	0.68431	0.60286	0.63018	0.58685
PhI-PPI-PPI	0.20543	0.21012	0.2429	0.21495
PhI-TRI-TRI	0.47729	0.482	0.49293	0.4964
PhI-TRI-PhI	0.74547	0.73301	0.76003	0.75558
PhI-TRI-PPI	0.72332	0.74558	0.69379	0.69289
PhI-PhI-PPI	0.68216	0.71124	0.68313	0.68548
PhI-PhI-PhI	0.57129	0.55712	0.64614	0.56509
PhI-PhI-TRI	0.33191	0.31186	0.34981	0.32088
TRI-PPI-TRI	0.42726	0.7594	0.27974	0.080837
TRI-PPI-PhI	0.50169	0.50245	0.52035	0.52855
TRI-PPI-PPI	0.099983	0.028912	0.74296	0.019359
TRI-TRI-TRI	0.40249	0.14744	0.28052	0.073205
TRI-TRI-PhI	0.47173	0.23071	0.022661	0.00078528
TRI-TRI-PPI	0.29195	0.067938	0.24517	0.023512
TRI-PhI-PPI	0.9191	0.53997	0.062653	0.88362
TRI-PhI-PhI	0.67232	0.70691	0.67418	0.69808
TRI-PhI-TRI	0.42338	0.19146	0.00020266	0.033381

Table S7: P-values for overrepresented regulatory path motifs in overexpression data with different cutoffs on transcriptional data

regulatory path	TRI(C)-h	TRI(C)-m	TRI(C)-l	TRI(M)
TRI	1.5551×10^{-12}	1.0955×10^{-13}	6.6975×10^{-19}	4.8242×10^{-94}
PPI	0.30853	0.31239	0.29239	0.32754
PhI	-	-	-	-
TRI-TRI	1.7648×10^{-4}	2.7434×10^{-7}	3.0133×10^{-8}	2.3561×10^{-21}
TRI-PPI	0.7682	0.79937	0.77587	0.77098
PPI-TRI	1.8191×10^{-6}	1.21×10^{-7}	8.3273×10^{-9}	5.4689×10^{-19}
PPI-PPI	0.69653	0.73384	0.69676	0.71979
TRI-PhI	0.59791	0.63614	0.6009	0.5585
PhI-TRI	-	-	-	-
PhI-PhI	-	-	-	-
PhI-PPI	-	-	-	-
PPI-PhI	0.14361	0.1523	0.15434	0.17426
PPI-PPI-TRI	0.80926	0.85596	0.90062	0.21517
PPI-PPI-PhI	0.90006	0.90327	0.88919	0.90568
PPI-PPI-PPI	0.76158	0.77686	0.78593	0.78918
PPI-TRI-TRI	0.0012288	1.5824×10^{-7}	1.9014×10^{-5}	0.0048585
PPI-TRI-PhI	0.49953	0.51295	0.47418	0.50277
PPI-TRI-PPI	0.7971	0.8037	0.809	0.81478
PPI-PhI-PPI	0.56487	0.21484	0.045527	4.6054×10^{-2}
PPI-PhI-PhI	0.057547	0.59242	0.61902	0.14332
PPI-PhI-TRI	0.00016744	2.5956×10^{-5}	4.4032×10^{-5}	2.8462×10^{-23}
PhI-PPI-TRI	0.18643	0.19428	0.17428	0.22566
PhI-PPI-PhI	0.83423	0.81863	0.82808	0.82434
PhI-PPI-PPI	0.06805	0.047571	0.088422	0.16465
PhI-TRI-TRI	-	-	-	-
PhI-TRI-PhI	-	-	-	-
PhI-TRI-PPI	-	-	-	-
PhI-PhI-PPI	0.927	0.91354	0.93168	0.93292
PhI-PhI-PhI	0.76118	0.7589	0.7753	0.76009
PhI-PhI-TRI	0.25421	0.25948	0.25943	0.27056
TRI-PPI-TRI	8.891×10^{-6}	0.00019631	1.4673×10^{-5}	1.4357×10^{-15}
TRI-PPI-PhI	0.66876	0.15159	0.099141	0.94607
TRI-PPI-PPI	0.82434	0.82419	0.85126	0.85327
TRI-TRI-TRI	0.057547	0.59242	0.61902	0.14332
TRI-TRI-PhI	0.50993	0.5114	0.51678	0.50968
TRI-TRI-PPI	0.56487	0.21484	0.045527	4.6054×10^{-16}
TRI-PhI-PPI	0.38976	0.55091	0.22095	0.048172
TRI-PhI-PhI	0.88646	0.88646	0.89236	0.8836
TRI-PhI-TRI	8.1355×10^{-5}	6.1744×10^{-8}	5.8734×10^{-6}	5.7987×10^{-15}
